# COVID-19 Vaccination in Patients With Malignancy; A Systematic Review and Meta-Analysis of the Efficacy and Safety

**DOI:** 10.3389/fendo.2022.860238

**Published:** 2022-05-02

**Authors:** Seyed Alireza Javadinia, Kimia Alizadeh, Mohammad-Shafi Mojadadi, Fateme Nikbakht, Farzaneh Dashti, Maryam Joudi, Hadi Harati, James S. Welsh, Seyed Amir Farahmand, Fahimeh Attarian

**Affiliations:** ^1^ Non-Communicable Diseases Research Center, Sabzevar University of Medical Sciences, Sabzevar, Iran; ^2^ Department of Diagnostic Medicine & Pathobiology, College of Veterinary Medicine, Kansas State University, Manhattan, KS, United States; ^3^ Leishmaniasis Research Center, Sabzevar University of Medical Sciences, Sabzevar, Iran; ^4^ Department of Epidemiology, School of Health, Mashhad University of Medical Sciences, Mashhad, Iran; ^5^ Faculty of Medicine, Birjand University of Medical Science, Birjand, Iran; ^6^ Department of Pediatrics, School of Medicine, Zabol University of Medical Sciences, Zabol, Iran; ^7^ Pediatric Gastroenterology and Hepatology Research Center, Zabol University of Medical Sciences, Zabol, Iran; ^8^ Department of Radiation Oncology, Edward Hines Jr Veterans Administration (VA) Hospital and Loyola University Chicago Stritch School of Medicine, Chicago, IL, United States; ^9^ Student Research Committee, Sabzevar University of Medical Sciences, Sabzevar, Iran; ^10^ Department of Public Health, School of Health, Torbat Heydariyeh University of Medical Sciences, Torbat Heydariyeh, Iran

**Keywords:** COVID-19, SARS-CoV-2, COVID-19 vaccines, neoplasms, cancer, vaccine, seroconversion, meta-analysis

## Abstract

**Background:**

Data on the efficacy and safety of COVID-19 vaccines in patients with malignancy are immature. In this paper, we assessed the literature involving the use of COVID-19 vaccines in cancer patients and reported the seroconversion rates as the main outcome and severity of COVID-19 infection and side effects following COVID-19 vaccination as the secondary outcomes.

**Methods:**

A systematic review with meta-analysis was performed. Searches were conducted in electronic websites, databases, and journals, including Scopus, PubMed, Embase, and Web of Science from January 01, 2019, to November 30, 2021. Studies reporting data on the safety and efficacy of COVID vaccine in cancer patients using any human samples were included. The risk of bias was assessed using the NEWCASTLE-OTTAWA scale in the included studies.

**Results:**

A total of 724 articles were identified from databases, out of which 201 articles were duplicates and were discarded. Subsequently, 454 articles were excluded through initial screening of the titles and abstracts. Moreover, 41 studies did not report the precise seroconversion rate either based on the type of cancer or after injection of a second dose of COVID vaccine. Finally, 28 articles met all the inclusion criteria and were included in this systematic review. The overall seroconversion rates after receiving a second dose of COVID-19 vaccine, based on type of cancer were 88% (95% CI, 81%-92%) and 70% (95% CI, 60%-79%) in patients with solid tumors and hematologic malignancies, respectively.

**Conclusion:**

Overall, we conclude that vaccination against COVID-19 in patients with active malignancies using activated and inactivated vaccines is a safe and tolerable procedure that is also accompanied by a high efficacy.

## 1 Introduction

The coronavirus disease 2019 (COVID-19) pandemic has affected many cancer patients worldwide. Besides the impact on delivery of cancer care due to severe acute respiratory syndrome coronavirus 2 (SARS-CoV-2) pandemic, studies have shown that these patients have a higher risk of severe illness from COVID-19 and exhibit poorer outcomes following COVID-19 treatments (1–3).

COVID-19 vaccination is presently the only available and valid option to overcome the pandemic considering the lack of effective treatment and the emerging coronavirus variants, even in patients with malignancies suffering from compromised immune responses (4–7). After introducing the first COVID-19 vaccines, patients with cancer have been prioritized for vaccination. Subsequently, numerous cohort and cross-sectional articles reporting the safety and the efficacy of the COVID-19 vaccines have been published. Due to the absence of a clinical trial and various scales to report the efficacy of vaccination, data on the efficacy and safety of COVID-19 vaccines in patients with malignancies are immature. Cancer patients were excluded from many of the clinical trials of COVID-19 vaccination. In this paper, we assessed the literature involving the use of COVID-19 vaccines in cancer patients and reported the seroconversion rates as the main outcome, with severity of COVID-19 infection and side effects following COVID-19 vaccination as the secondary outcomes.

## 2 Methods

This study is the first meta-data analysis to assess the safety and efficacy of COVID vaccine in cancer patients based on available studies published within the last 2 years. This study was conducted under the Guideline of Preferred Reporting Items Systematic Meta-Analyses Checklist (PRISMA) (8). At the beginning of this systematic review, all articles extracted from various databases were transferred to Endnote software.

### 2.1 Search Strategy

The online search was conducted by two separate authors (S.A.J. and F.A.) from electronic websites, databases, and journals, including Scopus, PubMed, Embase, and Web of Science from January 01, 2019, to November 30, 2021. Additionally, we manually screened references or citations of each article. The keywords used as a search term in this database were: [cancer patients [title] OR solid tumors [title] OR hematological malignancies [title] OR malignancy [title] OR chemotherapy [title] OR radiotherapy [title] OR immunotherapy [title] OR targeted therapy [title]] AND [COVID-19 [title] OR SARS-CoV-2 [title] OR COVID-19 vaccines [title] OR RNA vaccines [title] OR adenovirus-based vaccines [title] OR protein-based vaccines [title] OR inactivated coronavirus vaccines [title] OR seroconversion [title] OR side effects [title]]. After the primary search based on “search terms”, we removed duplicate studies.

### 2.2 Inclusion Criteria

The eligibility criteria for inclusion in this study were the following: (a) Randomized controlled trial (RCT) or Cohort Study design and (b) studies on cancer patients.

### 2.3 Exclusion Criteria

**Figure 1:**
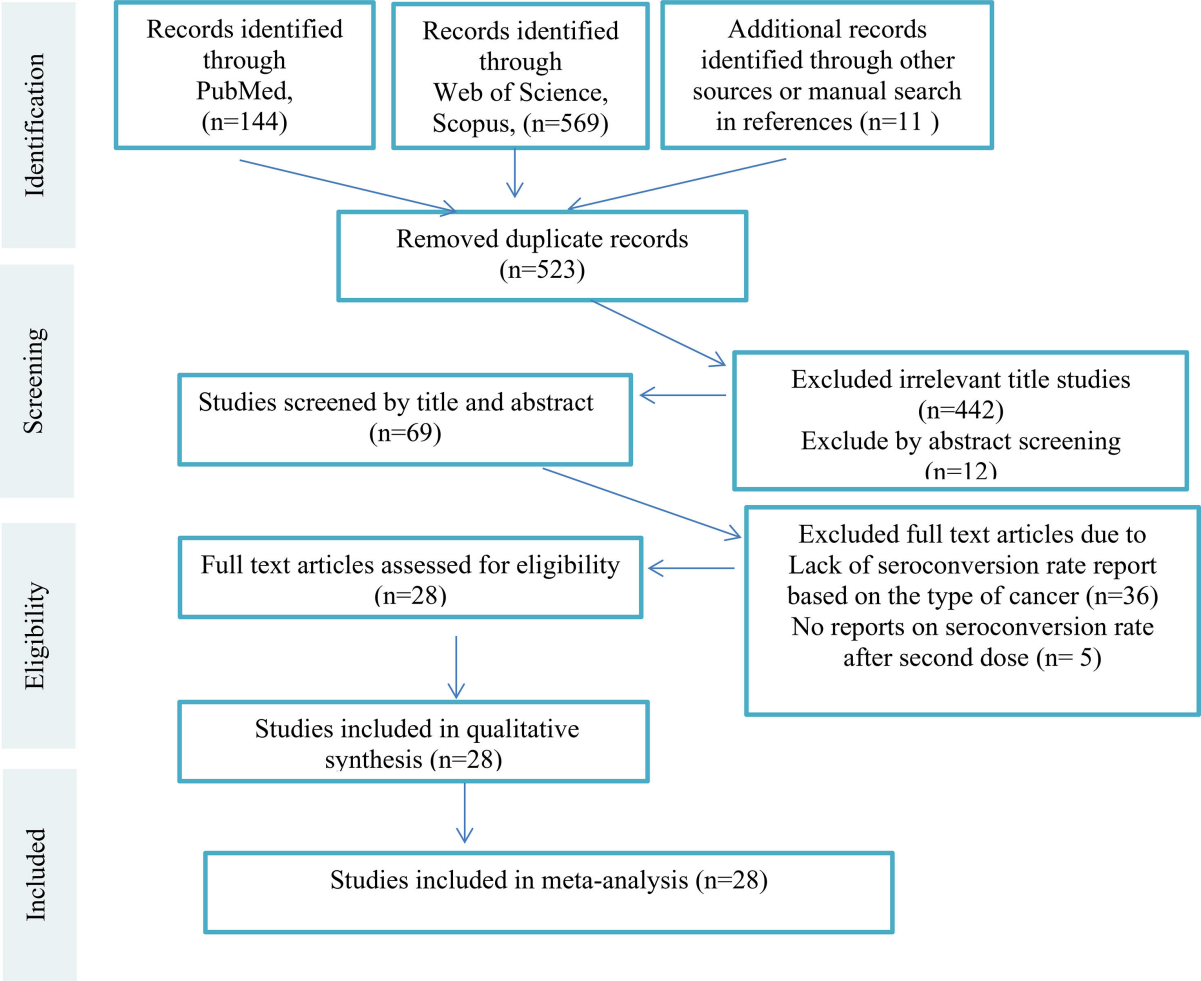
PRISMA Flow Diagram.

We excluded studies that were: (a) Animal studies (b) Evaluating non-cancer patients or healthy people (c) Reviews and book chapters, and (d) Articles written in any languages other than English. Editorials, commentary papers, and presentation reports could be included if they reported the seroconversion rates and prevalence of side effects rather than the author(s)’ opinion only. Then all remaining studies were screened by titles and abstract and irrelevant studies were excluded. Subsequently, full text versions of the remaining articles were assessed for eligibility independently by two authors (S.A.J. and F.A.). The third researcher (K.A.) evaluated the accuracy of selection of studies independently in all steps. All steps of study selection (PRISMA Flow Diagram) are shown in **Figure 1** [**Supplementary Material (Data Sheet 1)**].

### 2.4 Quality Assessment

The quality of eligible studies was evaluated using the NEWCASTLE-OTTAWA scale (9). This scale is a good measure of evaluation of the possible risk of bias in individual studies and quality assessment of cohort studies. Each study could be awarded 3 or more “stars” by S.A.J. and F.A. who read each article and completed the checklists separately. Stars were awarded in the representative selection sample, comparability between groups and completeness. Finally unbiased studies entered the meta-analysis (10). Provided that the results were discordant, the third author (K.A.) completed the checklist and the best matching results were selected (**Table 1**).

**Table 1:** Methodological quality summary for each included study.

Col	First author	Quality assessment
**1**	Mahil et al. (11)	*****
**2**	Ollila et al. (12)	*****
**3**	Roeker et al. (13)	****
**4**	Shah et al. (14)	****
**5**	Cavanna et al. (15)	*******
**6**	Monin et al. (16)	********
**7**	Shmueli et al. (17)	******
**8**	Gounant et al. (18)	******
**9**	Perry et al. (19)	*******
**10**	Herishanu et al. (20)	*******
**11**	Massarweh et al. (21)	*******
**12**	Pimpinelli et al. (22)	*******
**13**	van Oekelen et al. (23)	*****
**14**	Karacin et al. (24)	*****
**15**	Ariamanesh et al. (7)	****
**16**	Addeo et al. (25)	*****
**17**	Pimpinelli et al. (26)	***
**18**	Oosting et al. (27)	******
**19**	Webber et al. (28)	*****
**20**	Barrière et al. (29)	****
**21**	Thakkar et al. (30)	******
**22**	Grinshpun et al. (31)	*****
**23**	Goshen-Lago et al. (32)	******
**24**	Waldhorn et al. (33)	******
**25**	Agha et al. (34)	*****
**26**	Revon-Riviere et al. (35)	*****
**27**	Malard et al. (36)	****
**28**	Ram et al. (37)	****

*The result of quality assessment based on the NEWCASTLE-OTTAWA scale.

### 2.5 Data Extraction

The main variables in the present study include study design, authors’ name, publication months/year, type of the cancers and treatments administered, the stage of disease, type of COVID-19 vaccine (messenger RNA vaccines, adenovirus-based vaccines, protein-based vaccines and inactivated vaccines), number of doses, follow-up duration, clinical and serological outcomes, and side-effects. The data were extracted by S.A.J., F.D and F.A for each study. The fourth researcher (K.A.) evaluated the accuracy of selection studies independently in all steps.

### 2.6 Statistical Analysis

**Figure 2:**
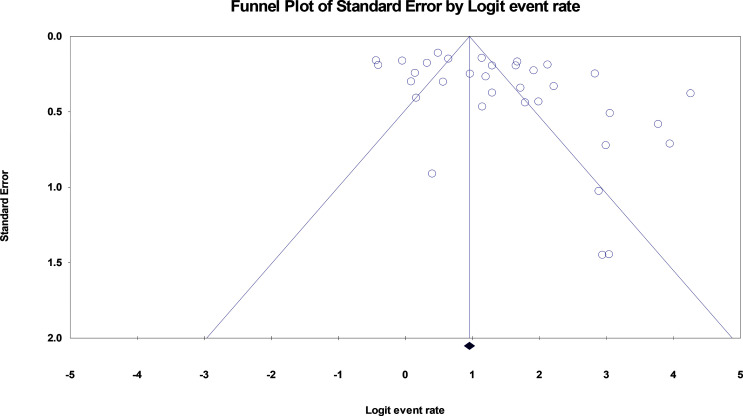
funnel plot of standard error by logit event rate. The Egger’s test was used, SE 3.7 (95% CI, 1.03, 6.37), t-valu82, df 32, P = 0.008 indicating a strong publication bias across the articles.

We used the reported overall point estimation of seroconversion rate after more than 2 weeks of the injection of COVID-19 vaccine (second dose), as a measure of immunogenicity of COVID 19 vaccine. We extracted seroconversion rate and confidence intervals (CIs) for studies that provided them separately, then we reported overall seroconversion rate and confidence intervals (CIs) by use of the assumptions of a random-effects model (which considers both within-study and between-study variations) as a measure of immunogenicity of COVID-19 vaccine in cancer patients. The rate of seroconversion estimation from random effects models were used, which incorporates between-study variability, since the tests for heterogeneity were statistically significant in all analyses. Statistical heterogeneity was evaluated with Cochran’s Q test and quantified by the I ^2^ statistic (38). To assess sources of heterogeneity, we conducted a subgroup analysis. Publication bias was assessed by visual inspection of funnel plot (**Figure 2**) (39). Statistical analyses were carried out with Comprehensive Meta-Analysis software, Version 3. A value of p<0.05 was considered statistically significant. All statistical tests were two-sided.

## 3 Results

The study flowchart (**Figure 1**) summarizes the searching process. A total of 724 articles were identified from databases, including Scopus, PubMed, Embase, Web of Science, and ScienceDirect out of which 201 articles were duplicates and discarded. Of the remaining 523 articles, 454 articles were unrelated and excluded by screening title and abstract, 69 full text articles were assessed for eligibility, 41 studies did not have an exact report of seroconversion rate based on type of cancer or did not report seroconversion rate after injection of the second dose of COVID-19 vaccine. Among the 28 remaining studies, 4 were letters and 24 were original studies. Finally, 28 articles met all the inclusion criteria and were included in this systematic review (**Table 1**). **Table 2** summarizes the available clinical data (number of patients, disease characteristics, treatment details and outcomes) from the 28 studies that used COVID-19 vaccines in cancer patients. The clinical data evaluated includes 24 cohort studies (17 prospective studies, 11 retrospective studies).

**Table 2:** Characteristics of enrolled studies to meta-analysis.

	First Author, Month/year	Country	Patients (n)	Female/Male	Age >18 median	Type of Cancer	COVID-19 Vaccine (2 doses)	Median Follow up after second dose
1	Mahil et al., Oct 2021 (11)	Italy	88	23/65	68 (61.5-73)	Solid tumor	BNT162b2	21 days
2	Ollila et al., Nov 2021 (12)	Island	160	74/86	72 (65–79)	Hematologic	BNT162b2 & mRNA-1273	N/M
							Johnson & Johnson	
3	Roeker et al., May 2021 (13)	USA	44	21/23	71 (37-89)	Hematologic	BNT162b2 & mRNA-1273	21 days
4	Shah et al., Nov 2021 (14)	NA	89	51/38	NA	Hematologic	BTN162b26 & mRNA-1273	14 days
5	Cavanna et al., Sep 2021 (15)	Italy	257	144/113	65 (57–72)	Solid tumor	BNT162b2 & mRNA-1273	28 days
6	Monin et al., Aug 2021 (16)	UK	24	NA	73 (64.5-79.5)	Solid & Hematologic tumors	BNT162b2	12 weeks
7	Shmueli et al., Sep 2021 (17)	Israel	129	67/62	62 (32-84)	Solid tumor	BNT162b2	N/M
8	Gounant et al., Nov 2021 (18)	French	325	181/125	67 (27-92)	Solid tumor	BNT162b2	2-9 weeks
9	Perry et al., Aug 2021 (19)	Israel	149	61/88	64 (1)	Hematologic	BNT162b2	14-21 days
10	Herishanu et al., Jun 2021 (20)	Israel	167	55/112	71 (1)	Hematologic	BNT162b2	15 days
11	Massarweh et al., May 2021 (21)	Israel	102	44/58	66 (64-80)	Solid tumor	BNT162b2	3- 54 days
12	Pimpinelli et al., May 2021 (22)	Italy	92	43/49	NA	Hematologic	BNT162b2	N/M
13	Van Oekelen et al., Aug 2021 (25)	USA	260	135/185	68 (1)	Hematologic	BNT162b2	N/M
14	Karacin et al., Aug 2021 (24)	Turkey	47	18/29	73 (64–80)	Solid tumor	inactivated vaccine	4 weeks
15	Ariamanesh et al., Oct 2021 (7)	Iran	364	217/147	54 (18–85)	Solid tumor	BBIBP-CorV	N/M
16	Addeo et al., August 2021 (25)	US/Europe	131	59/72	63 (55–69)	Solid & Hematologic tumors	BNT162b2&mRNA-1273	N/M
17	Pimpinelli, et al. July 2021 (26)	Italy	42	NA	NA	Hematologic	BNT162b2	N/M
18	Oosting et al., Dec 2021 (27)	Netherlands	503	260/243	NA	Solid tumor	mRNA-1273	N/M
19	Webber et al., Dec 2021 (28)	Italy	291	173/118	68.2 (60–75)	Solid tumor	BNT162b2	21 days
20	Barrière et al., April 2021 (29)	France	42	NA	NA	Solid tumor	BNT162b2	N/M
21	Astha Thakkar, Aug 2021 (30)	USA	200	116/84	67 (27–90)	Solid & Hematologic tumors	BNT162b2&mRNA-1273	30 days
							AD26.COV2. S	
22	A. Grinshpun, Sep 2021 (31)	Israel	172	NA	62 (23–91)	Solid tumor	BNT162b2	77 days
23	Tal Goshen-Lago, July 2021 (32)	Israel	232	100/132	NA	Solid & Hematologic tumors	BNT162b2	14 days
24	Ithai Waldhorn, Oct 2021 (33)	USA	154	70/884	67 (32–87)	Solid tumor	BNT162b2	N/M
25	Mounzer Agha, Jan 2021 (34)	France	67	32/35	65 (57–72)	Hematologic	BNT162b2 & mRNA-1273	23 days
26	Gabriel Revon-Riviere, June 2021 (35)	Israel	13	11/2	NA	Solid tumor	BNT162b2	N/M
27	Florent Malard, Aug 2021 (36)	French	195	117/78	69 (21.5-91.7)	Hematologic	BNT162b2	14 days
28	Ron Ram, June 2021 (37)	Israel	80	36/44	65 (23–83)	Hematologic	BNT162b2	N/M

N/M, Not mentioned; NA, Not available.

**Figure 3:**
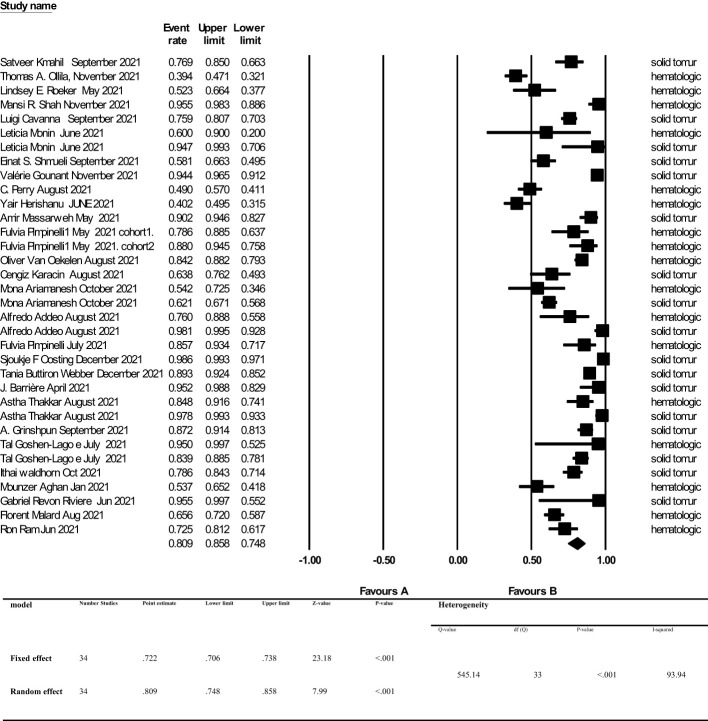
Seroconversion rate after more than 2 weeks of receiving second dose of COVID-19 vaccine in cancer patients. Addeo et al. (25), Agha et al. (34), Ariamanesh et al. (7), Barrière et al. (29), Cavanna et al. (15), Goshen-Lago et al. (32), Gounant et al. (18), Grinshpun et al. (31), Herishanu et al. (20), Karacin et al. (24), Mahil et al. (11), Malard et al. (36), Massarweh et al. (21), Monin et al. (16), Ollila et al. (12), Oosting et al. (27), Perry et al. (19), Pimpinelli et al. (22), Pimpinelli et al. (26), Ram et al. (37), Revon-Riviere et al. (35), Roeker et al. (13), Shah et al. (14), Shmueli et al. (17), Thakkar et al. (30), van Oekelen et al. (23), Waldhorn et al. (33), Webber et al. (28).

**Figure 4:**
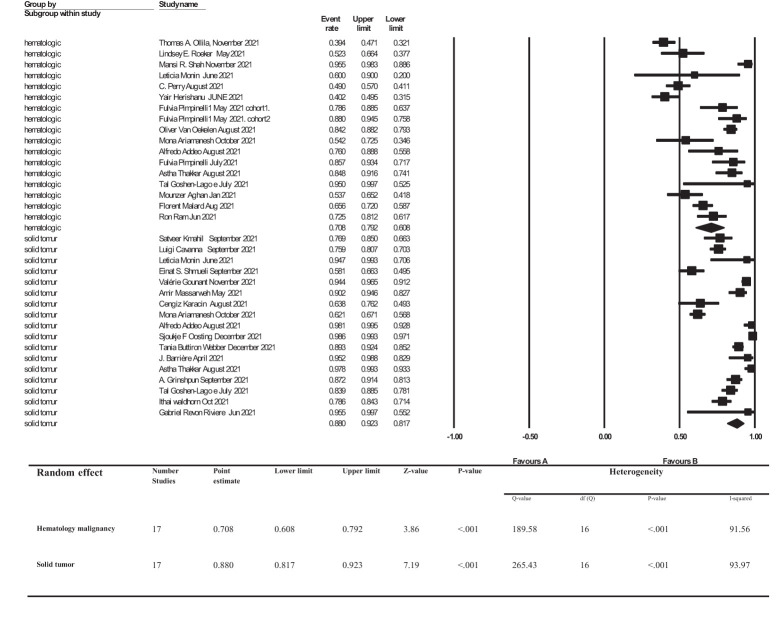
subgroup analysis of Seroconversion rate after more than 2 weeks of receiving second dose of COVID-19 vaccine in cancer patients. Addeo et al. (25), Agha et al. (34), Ariamanesh et al. (7), Barrière et al. (29), Cavanna et al. (15), Goshen-Lago et al. (32), Gounant et al. (18), Grinshpun et al. (31), Herishanu et al. (20), Karacin et al. (24), Mahil et al. (11), Malard et al. (36), Massarweh et al. (21), Monin et al. (16), Ollila et al. (12), Oosting et al. (27), Perry et al. (19), Pimpinelli et al. (22), Pimpinelli et al. (26), Ram et al. (37), Revon-Riviere et al. (35), Roeker et al. (13), Shah et al. (14), Shmueli et al. (17), Thakkar et al. (30), van Oekelen et al. (23), Waldhorn et al. (33), Webber et al. (28).

The overall point estimation of seroconversion after more than 2 weeks beyond the second dose of COVID-19 vaccine injection was 81% (95% CI, 75%-86%) (Random effect), although heterogeneity between studies was significant (I^2 =^ 93%, P _heterogeneity_<0.001) (**Figure 3**). In subgroup meta-analyses conducted by the type of cancer, the overall seroconversion rate after receiving the second dose of COVID-19 vaccine-based type of cancer were 88% (95% CI 81%-92%) and 70% (95% CI, 60%-79%) in patients with solid tumor and hematologic malignancy, respectively. Heterogeneity between subgroups categorizing the types of cancer was also significant (I^2 =^ 93.9%, P _heterogeneity_<0.001 and I^2 =^ 91.5%, P _heterogeneity_<0.001, respectively) (**Figure 4**).

## 4 Discussion

In this review and analysis, we assessed the literature involving the use of COVID-19 vaccines in cancer patients and reported the seroconversion rates, clinical COVID-19 infection rates, severity of COVID-19 infection and side effects following COVID-19 vaccination.

### 4.1 Efficacy of COVID-19 Vaccination

The overall seroconversion rate beyond 2 weeks after the second dose of a COVID-19 vaccine was 81%. However, we found that the seroconversion rate was significantly higher in patients with solid tumors compared to patients with hematologic malignancies [88% (95% CI, 81%-92%) vs 70% (95% CI, 60%-79%), respectively].

#### 4.1.1 Factors Affecting the Seroconversion Rates After COVID-19 Vaccination in Patients With Cancer

We observed evidence of seroconversion and antibody production against the SARS-CoV-2 virus in about 80% of cancer patients who were vaccinated. Nevertheless, the intensity of humoral response depends on several factors including type of malignancy (solid tumors versus hematologic malignancies), type of cancer treatment administered, demographic characteristics of the patient and other underlying factors, which will be discussed in the following sections.

##### 4.1.1.1 Type of Malignancy

In the studies which only investigated patients with solid tumors (n=17), authors in general reported a seroconversion rate of 0.817 to 0.923. Moreover, in the studies exclusively on hematologic malignancies (n=11) seroconversion rates were between 0.608 and 0.792. Finally, in studies performed by Monin et al. (16), Pimpinelli et al. (22, 26), Ariamanesh et al. (7), Addeo et al. (25) and Thakkar et al. (30) on patients with both solid tumors and hematologic malignancies, the seroconversion rate was significantly lower in patients with hematologic malignancies (60-81.7% vs 93.8-98%). A proper immune response to either vaccines or viral infections requires both cellular and humoral immunity. An adequate antibody response can, in turn, increase T lymphocyte activity (CD4+ T helper) and lead to higher stimulation of B lymphocytes to produce higher levels of antibodies (20, 40, 41). It has been shown that there is a correlation between CD4+ T helper cell response to viral spike protein and anti-SARS-CoV-2 IgG and IgA titer levels (42–46). In general, based on our findings, it can be concluded that seroconversion rates in patients with hematologic malignancies are lower than in patients with solid tumors. It has been previously shown that, compared to patients with solid tumors and healthy individuals, in patients with hematologic malignancies, T cell immune responses are reduced both after COVID-19 infection and post-vaccination, with CD4+ T cells playing a principal role in comparison to CD8+ (24, 32, 35–37, 40). Furthermore, patients with hematologic malignancies undergo somewhat more significant immunosuppression, mainly due to cancer-induced immune dysregulation or because they receive therapies that can provoke myelosuppression and lymphodepletion. These factors collectively are not only involved in a greater risk for infection and mortality, but also a poorer response to vaccination (47–51). Our findings, indicating differences in immune responses based on type of malignancy, are in agreement with previous publications investigating vaccination-based immune responses against seasonal influenza (52, 53).

##### 4.1.1.2 Type of Treatment

In the studies performed by Cavanna et al., (15) (15 seroconversion to 22, 68.18%), Addeo et al., (25) (13 to 14, 93%) and Oosting et al., (27) (122 to 131, 93%) on cancer patients receiving only immunotherapy as their anticancer therapeutic regimen, seroconversion rate was 68.18-93%. Additionally, in patients only receiving chemotherapy investigated in the studies by Oosting et al., (27), (192 of 229, 84%), Webber et al., (28) (99 to 115, 86%) and Grinshpun et al., (31), (81.3%) seroconversion rates of 81.3-86% were reported. Although vaccinated patients receiving immunotherapy comprise a small portion of most of previous researches, it appears that in patients who underwent immunological manipulation, seroconversion rate is weakened. As an example, it has been previously reported that active treatment with B cell-depleting agents such as rituximab and obinutuzumab (anti-CD20), or plasma cell depleting-agents such as daratumumab (anti-CD38) can decrease antibody production following COVID-19 vaccination, especially in patients with lymphoid malignancies. This is mainly a consequence of peripheral B cell depletion and B cell signaling pathway disruption caused by anti-CD20 agents, and anti-CD38-induced reduction of normal plasma cells, followed by a decrease in polyclonal immunoglobulin levels which are vital for humoral immunity (18, 22, 47, 49, 54–57). Furthermore, impaired response to vaccination induced by anti-CD20 therapy in patients with cancer has been similarly reported in the case of influenza or *Streptococcus pneumonia* vaccines (52, 58).

##### 4.1.1.3 Other Factors

In addition to the type of malignancy and anticancer treatment, patient’s demographic factors such as age and gender might also contribute to the rate of seroconversion in response to vaccination against COVID-19. Ma et al. have shown that age is the most prominent predicting factor with regards to the failure of anti-COVID-19 vaccine in cancer patients and negatively correlates with seroconversion rate in these individuals (59). These results are consistent with another study on BNT162b2 vaccine which shows a remarkable difference between the seroconversion rate of young, versus elderly cancer patients, with the titer levels also being significantly different (32).

Gender has been shown to be another contributing factor in seroconversion rate among patients with cancer. In a study on seroprevalence against COVID-19 in cancer patients, Javadinia et al. has shown that seropositivity against COVID-19 is greater in females, with higher rates in patients with breast cancer and gynecologic cancers (60). Female hormones are known to enhance the immune system, and sex steroids have been shown to correlate with different immune responses to COVID-19 (61, 62).

### 4.2 Safety of COVID-19 Vaccination

Studies have shown that COVID-19 vaccination in patients with malignancy is safe and tolerable regardless of their types. Both systemic and locoregional side-effects including fatigue, hypersensitivity reactions, fever, asthenia, vomiting, headaches, muscle and joint pain, chills, transient lymphadenopathy and injection site reactions (pain, erythema, itchiness, swelling, and redness) were reported. Although the various instruments and methods which were used for assessment of frequency, severity, and extend of the side-effects make it difficult to perform a meta-analysis investigation in this context, most of reported vaccines related side-effects were grade II, maximally (7, 11, 15, 17, 19, 20, 22, 24, 28, 32, 35–37).

### 4.3 Limitations

This meta-analysis harbors a strong publication bias which we believe is due to the high verity of published articles’ methodologies and extent of factors affecting the immune response in this population [age, type of cancer (solid tumors and hematologic malignancies) or status of treatment (receiving chemotherapy or bone marrow transplant vs radiotherapy or follow-up)]. Moreover, not reporting the exact prevalence of PCR-confirmed COVID-19 infection and the its outcome is another important limitation of previous studies affecting the results of present review.

## 5 Conclusion

In general, we can conclude that vaccination against COVID-19 in patients dealing with malignancies is a safe and tolerable procedure, which is also accompanied by a high efficacy. Nevertheless, a number of factors such as type of malignancy, type of anti-cancer therapy administered, and demographic factors of the patient can influence the efficiency of anti-covid vaccination in these patients. There are a couple of suggestions to improve the efficiency of anti-covid vaccination in cancer patients, particularly patients with hematologic malignancies, and subsequent to receiving immunosuppressive anti-cancer therapies such as anti-CD20 or anti-CD38 therapies. These patients can take advantage of passive immunization using anti-COVID-19 antibodies. Moreover, booster doses or higher doses of some vaccines, or taking benefit from mixed vaccine types are suggested in these individuals. Additional studies are required to investigate the efficacy of the aforementioned approaches in these patients, especially in the individuals who were vaccinated in the course of active treatment.

## Data Availability Statement

The original contributions presented in the study are included in the article/**Supplementary Material**. Further inquiries can be directed to the corresponding author/s.

## Ethics Statement

The Ethic Committee of Sabzevar University of Medical Sciences approved the study protocol (ID IR.MEDSAB.REC.1400.148).

## Author Contributions

Conceptualization; SJ and SF. Data curation; KA and FA. Funding acquisition; SJ. Investigation; FA and M-SM. Methodology; FA, FN, and FD. Project administration; SJ. Software; FA, SJ, FN, and FD. Supervision; SJ and JW. Validation; FA and HH. Visualization; SJ, FA. Roles/Writing original draft; FA, SJ, and KA. Writing - review & editing; FA, SJ, M-SM, and KA. All authors contributed to the article and approved the submitted version.

## Funding

Sabzevar University of Medical Sciences (grant number 400237).

## Conflict of Interest

The authors declare that the research was conducted in the absence of any commercial or financial relationships that could be construed as a potential conflict of interest.

## Publisher’s Note

All claims expressed in this article are solely those of the authors and do not necessarily represent those of their affiliated organizations, or those of the publisher, the editors and the reviewers. Any product that may be evaluated in this article, or claim that may be made by its manufacturer, is not guaranteed or endorsed by the publisher.
